# Gait Analysis with Wearables Can Accurately Classify Fallers from Non-Fallers: A Step toward Better Management of Neurological Disorders

**DOI:** 10.3390/s20236992

**Published:** 2020-12-07

**Authors:** Rana Zia Ur Rehman, Yuhan Zhou, Silvia Del Din, Lisa Alcock, Clint Hansen, Yu Guan, Tibor Hortobágyi, Walter Maetzler, Lynn Rochester, Claudine J. C. Lamoth

**Affiliations:** 1 Translational and Clinical Research Institute, Faculty of Medical Sciences, Newcastle University, Newcastle Upon Tyne NE4 5PL, UK; Silvia.Del-Din@newcastle.ac.uk (S.D.D.); Lisa.Alcock@newcastle.ac.uk (L.A.); Lynn.Rochester@newcastle.ac.uk (L.R.); 2 Department of Human Movement Sciences, University Medical Center Groningen, University of Groningen, 9713 AV Groningen, The Netherlands; y.zhou01@umcg.nl (Y.Z.); t.hortobagyi@umcg.nl (T.H.); c.j.c.lamoth@umcg.nl (C.J.C.L.); 3 Department of Neurology, University Hospital Schleswig-Holstein, Campus Kiel, 24105 Kiel, Germany; c.hansen@neurologie.uni-kiel.de (C.H.); w.maetzler@neurologie.uni-kiel.de (W.M.); 4 School of Computing, Newcastle University, Newcastle Upon Tyne NE4 5TG, UK; yu.guan@ncl.ac.uk; 5 Newcastle upon Tyne Hospitals NHS Foundation Trust, Newcastle Upon Tyne NE7 7DN, UK

**Keywords:** neurological disorders, machine learning, classification, fall, path signature, gait, inertial measurement unit, data pre-processing, fall risk assessment, wearables

## Abstract

Falls are the leading cause of mortality, morbidity and poor quality of life in older adults with or without neurological conditions. Applying machine learning (ML) models to gait analysis outcomes offers the opportunity to identify individuals at risk of future falls. The aim of this study was to determine the effect of different data pre-processing methods on the performance of ML models to classify neurological patients who have fallen from those who have not for future fall risk assessment. Gait was assessed using wearables in clinic while walking 20 m at a self-selected comfortable pace in 349 (159 fallers, 190 non-fallers) neurological patients. Six different ML models were trained on data pre-processed with three techniques such as standardisation, principal component analysis (PCA) and path signature method. Fallers walked more slowly, with shorter strides and longer stride duration compared to non-fallers. Overall, model accuracy ranged between 48% and 98% with 43–99% sensitivity and 48–98% specificity. A random forest (RF) classifier trained on data pre-processed with the path signature method gave optimal classification accuracy of 98% with 99% sensitivity and 98% specificity. Data pre-processing directly influences the accuracy of ML models for the accurate classification of fallers. Using gait analysis with trained ML models can act as a tool for the proactive assessment of fall risk and support clinical decision-making.

## 1. Introduction

The world population is getting older and the risk of falling increases with age [1]. One third of adults over 65 experience at least one fall each year [2] and this proportion increases with age [3]. Falls can lead to severe fatal and nonfatal injuries [4] and are associated with mortality, morbidity and a poor quality of life in older adults [5]. People with neurological disorders fall more often compared to healthy adults of a similar age [6], and this can increase their physiological, psychological and financial burden [7]. Therefore, it is crucial to identify people with neurological disorders at risk of falls, before a fall occurs, so that interventions are offered early.

Extrinsic (e.g., weather, lighting, uneven surfaces) and intrinsic (e.g., cognition, vision, muscle strength, gait) factors can predispose individuals to falls [8,9,10]. Extrinsic factors are difficult to control; however, intrinsic factors can be mitigated with appropriate interventions [11]. Among them, the strongest independent intrinsic fall risk factors are physical weakness, gait and balance impairments, psychoactive medications and previous falls [12,13]. Dizziness as well as visual and cognitive impairment also play a role [10,14,15,16]. Gait speed is considered as a marker of global health, and by evaluating gait using instrumented assessments, it is possible to assess individual fall risk [17,18]. Early detection of fall risk is an essential component of effective fall prevention in older adults to reduce the risk of future falls [1,8].

In clinical settings, fall risk assessment has evolved from a simple questionnaire to functional tests such as the timed up and go [19] and the Berg Balance Scale [20]. These tests provide a good indication of mobility; however, they are poor predictors of future falls [21]. As the majority of falls occur while walking [22], functional tests are unable to assess dynamic characteristics of gait (such as step velocity, step length, step time, variability and asymmetry), i.e., the spatial-temporal characteristics of gait. With recent advances in microelectromechanical systems, wearable sensors are cost effective, and can give accurate, objective and quantifiable dynamic gait characteristics [23,24]. Due to their small size, portability, high storage capacity and long battery life, they are ideal for continuous monitoring of gait for fall risk assessment [25,26].

Wearables have been used to assess gait in clinical and free-living conditions [23,24,25,26,27,28,29]. Gait characteristics obtained through signal-processing methods can be used to characterise fallers and non-fallers and these outcomes may be used to inform tailored intervention rehabilitation plans [30]. Fallers with neurological disorders (e.g., Parkinson’s disease (PD)) showed higher variability in gait rhythmicity, higher asymmetry and a slower pace compared to non-fallers [31,32]. Gait characteristics measured with wearable devices can be used to train predictive models for accurate fall risk assessment [27,28]. However, methods for appropriately utilising this information for accurate fall risk assessment in people with neurological disorders are yet to be established.

Various traditional supervised machine learning (ML) models, such as random forest (RF), support vector machine (SVM), k-nearest neighbour (KNN), Naïve Bayes (NB), logistic regression (LR), decision tree (DT), linear discriminant analysis and others, have been used to classify older adult fallers from non-fallers with a classification accuracy between 69% and 100% [27,33,34,35,36]. All these studies involved healthy older adults or focussed on one patient group. Only one study [28] has considered a heterogeneous group of neurological disorders and reported maximum classification area under the curve (AUC) of 0.77 to distinguish fallers from non-fallers. For accurate fall prediction, classification accuracy needs to be improved.

The accuracy of fall classification models is reduced due to heterogeneity of the data. For example, the underlying distribution of gait characteristics from different neurological conditions (such as PD, dementia, stroke and others) is important. Classifiers such as linear discriminant analysis may not work well when independent characteristics do not follow a multivariate normal distribution [37], and data transformation may help improve the accuracy and generalisability of the classifier [38]. Similarly, KNN is an instance-based learner where performance is influenced by a greater number of independent gait characteristics. In contrast, RF models can handle a high number of correlated features. Consequently, principal component analysis (PCA) is often used to reduce data dimensionality and train models only on useful information [39]. It is also important to capture both linear and nonlinear interactions among the gait characteristics to train the machine learning (ML) models for better performance [40]. A path signature method is useful to evaluate nonlinear interactions and can be used to extract unique geometrical features from a stream of spatial-temporal gait data, based on the theory of rough path to train the classifiers for optimal classification accuracy [41]. It is common practice in ML to standardise or normalise datasets to meet the underlying assumptions of various classifiers and avoid the influence of input features upon scaling for improved classification. Pre-processing methods are required to transform the data so that ML models can learn the unexpected shift to testing data outside of the training distribution to increase classification accuracy [42,43].

Therefore, to extract representative features for the accurate classification of fallers, this study examined different data pre-processing methods. The specific aim of this study was to compare the effects of data pre-processing methods on the performances of ML models to optimise the classification of fallers and non-fallers for the better management of people with neurological disorders. Pre-processing methods such as standardisation, PCA and the path signature method were compared. We hypothesised that pre-processing methods will influence classification performance and that a model, which can reduce data dimensionality and consider both linear and nonlinear interactions, is likely to produce the most accurate classification of fallers and non-fallers.

## 2. Materials and Methods

### 2.1. Participants

In total 384 participants with neurological disorders were included in the analysis [44]. These patients were recruited between September 2014 and April 2015 from three neurology wards of the University Hospital of Tubingen, Tubingen, Germany [44]. Participants were included provided they were able to walk 20 m with or without a walking aid. Exclusion criteria were as follows: an inability to provide informed consent, a high risk of falls (defined as >1 fall per week) and severe cognitive impairment as defined by a Mini-Mental State Examination (MMSE) score ≤10 points [44]. Participants were classified as fallers if they had fallen at least once during a two-year period prior to recruitment. The study was approved by the Institutional Ethics Committee (No. 356/2014BO2), University Hospital of Tubingen, Tubingen, Germany, and all participants gave written informed consent prior to participation.

### 2.2. Gait Assessment

**Figure 1:**
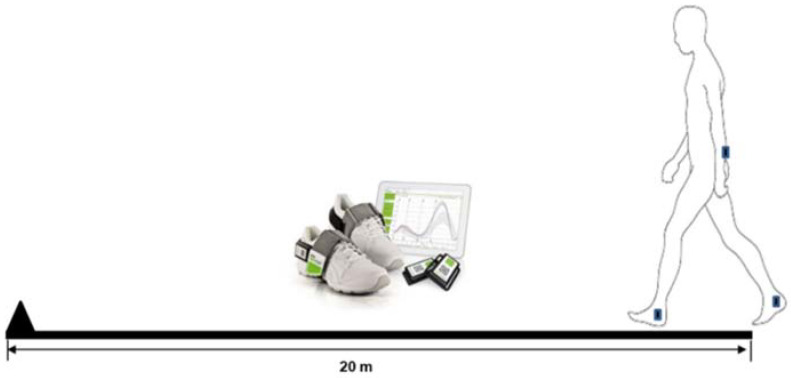
Protocol for gait assessment in the neurology ward.

Participants performed various tasks (gait and balance) and, among them, they were instructed to walk 20 m at a self-selected comfortable speed (Figure 1), which was selected for further analysis. An inertial measurement unit (IMU) wearable sensor system (Rehawatch^®^, Hasomed, Magdeburg, Germany) was attached with Velcro straps at the lower back (L4-L5) and at both ankles (placed laterally, superior to the malleoli) to collect data during walking at a sampling frequency of 100 Hz. The IMU system comprised a 3D accelerometer (range: ±8 g), 3D gyroscope (range: ±2000 °/s) and 3D magnetometer (range: ±1.3 Gs) [44,45].

### 2.3. Extraction of Gait Characteristics

From 384 participants, 349 were able to perform the described gait assessment (19% PD (fallers: 8%), 19% stroke (fallers: 5%), 11% epilepsy (fallers: 4%), 10% pain syndromes (fallers: 3%), 9% multiple sclerosis (fallers: 4%), 7% central nervous system (CNS) tumour (fallers: 2%), 6% vertigo (fallers: 2%), 6% dementia (fallers: 2%) and 6% meningitis/encephalitis (fallers: 1%)) [44]. A total of 27 gait characteristics were extracted from the RehaGait gait assessment system directly using the manufacturer’s inbuilt algorithms and data from foot sensors to identify gait cycles [45]. The RehaGait system is a low-cost complete hard- and software solution for clinical gait analysis. The assessor is guided through a predefined number of clinical assessments (e.g., 20 m walk, timed up and go, static balance) and, once the participant has finished the assessment, reports are created for further use. Results of each assessment were then extracted and used for further statistical analyses. In our case, no additional steps were incorporated as the system is a well-established and validated system for clinical gait and balance assessments [45]. Gait characteristics included the mean and standard deviation of stride duration, stride length, stride velocity, number of steps, stance and swing phase duration (also as a percent of the gait cycle), stance time, swing time, symmetry of stance and swing phases, single support time, heel-strike angle, toe-off angle, foot circumduction, as well as spatial and temporal variability in gait cycle (brief definitions are given in Appendix B). These variables have been shown to be accurate and valid [45] as well as sensitive to ageing and neurodegenerative diseases [46,47,48,49].

### 2.4. Statistical Analysis

Parametric (Student’s *t*-test) and nonparametric (Mann–Whitney) tests were performed on the extracted gait characteristics to evaluate the significant difference between fallers and non-fallers based on the normality test (Shapiro–Wilk). For further data exploration prior to method selection, correlation among the gait characteristics was investigated to check the linearity/nonlinearity and collinearity. To overcome the effect of high correlation, collinearity and dimensional space (large number of gait features), it was critical to carry out data pre-processing methods for better classification modelling.

### 2.5. Methods of Data Pre-Processing for Machine Learning Models

Each of the gait characteristics extracted had different scales. Various data processing methods such as technique standardisation PCA after standardisation and path signature analysis were implemented and compared to investigate their impact on the ML models.

#### 2.5.1. Standardisation

Standardisation of input features (gait characteristics) to ML models is important when extracted characteristics have different units (scales) [50], which can impact model performance due to their sensitivity to the scales of the characteristics. This sensitivity can be reduced by converting the input gait characteristics to z-scores for normalisation (zero mean, unit variance) with the following equation:

(1) $$z_{i}=\frac{x_{ij}-\bar{x_{i}}}{\sigma_{i}}$$

where $x_{ij}$ represents each gait characteristic, i is the individual value from each study participant j is used to convert into the $z_{i}$ score. $\bar{x_{i}}=\frac{1}{N}\overset{N}{\underset{j}{\sum }}\left(x_{j}\right)$ and $\sigma_{i}=\sqrt{\frac{1}{N}\overset{N}{\underset{j=1}{\sum }}{\left(x_{ij}-\bar{x_{i}}\right)}^{2}}$ are mean and standard deviation of each group (non-fallers and fallers). N represents the number of participants in each group.

#### 2.5.2. Principal Component Analysis 

To reduce the dimensional space and correlation among gait characteristics, PCA was used to extract significant useful information. Preserving maximum variance from a large number of gait characteristics is essential for efficient and better training of ML models [51,52]. Orthogonal bases created by PCA capture the maximum variance for the gait characteristics data, and PCA also creates the uncorrected coefficients’ expansion in the form of newly generated features [52].

PCA will convert the D-dimensional training set X having dimensional N× D into N× d. D in our case is 27 (number of gait characteristics) and d is the number of selected components from the PCA. The covariance matrix computation for PCA is based on the following equation:

(2) $$Cov\left(x\right)=\frac{1}{N}\sum_{j=1}^{N}(x_{i}-\bar{x}){\left(x_{i}-\bar{x}\right)}^{T}$$

Then, eigenvectors (***v*_1_**,***v*_2_**,...,***v_N_***) and corresponding eigenvalues (**λ_1_**,**λ_2_**,...,**λ_N_**) were computed based on the following equation:

(3) Cov(x)ν=λν

Finally, by sorting the eigenvectors in descending order, eigenvectors with the largest eigenvalues were selected to form an N × d dimensional matrix ***W*** (where every column represents an eigenvector). Transformed data (y) to new space are summarised with the following equation:

(4) $$y=W^{T}\times x$$

#### 2.5.3. Path Signature Method

The motivation to use this method is to generate new features that capture the linear and nonlinear interaction among gait characteristics. Classifiers from nonlinear domains, such as neural networks or RF, are considered to give a higher classification performance; however, their functions are difficult to understand. Therefore, with the path signature method, nonlinearity can be encoded to the extracted features to train simple classifiers for a higher performance and provide an interpretable solution [40].

The path signature method is based on differential equations driven by the theory of rough paths and is used to extract unique geometric features from the original dataset to train ML models for a better performance [41]. The rough path theory is related to the interaction between nonlinear systems [53]. The signatures of the continuous path that characterise its shape are the iterated integrals, which is an infinite sequence of numbers [54]. In other words, it is a generalisation of Taylor’s theorem to extract the signatures [55], which contain all the necessary information from the path for accurate prediction of the output class labels (fallers vs. non-fallers).

The systematic way to provide a feature set for sequential data is path signature [54,56]. The extracted gait characteristics in this study were used to make a two-dimensional path ${\mathbb{R}}^{2}$. Initially, the path signatures were defined for the continuous paths; however, they can be extended to discrete paths [57]. The signatures are independent from the choice of timescale used for interpolation in the case of discrete data.

**Figure A1:**
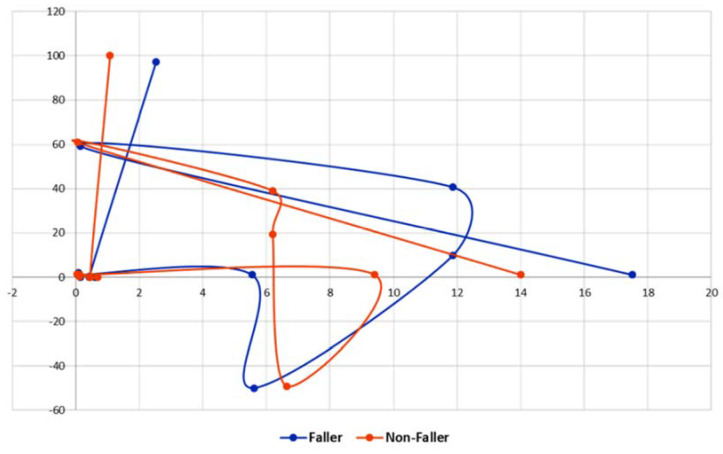
Piecewise linear path for signature extractions for training ML models.

In our case, the path of the dataset is piecewise linear (an example for this linear path is shown in Appendix C Figure A1). Thus, the integrals are non-essential for computing the signatures [58]. The entire signature path is achieved by first calculating the signature of its pieces and then joining them. The element of signature for each line path can be computed as follows [58]:

(5) $$S{\left(P\right)}_{t,t+1}^{i_{1},i_{2},\ldots ,i_{k}}=\frac{1}{k!}\overset{k}{\underset{j=1}{\prod }}\left(P_{t+1}^{i_{j}}-P_{t}^{i_{j}}\right)$$

where *P* indicates the signature path and $P_{t}^{i_{j}}$ represents the $i_{j}$-th coordinate of this path. For the entire path based on the gait characteristics vector from each subject, the signature of the path at any time stamps (s,t,u) satisfying the conditions s<t<u according to the Chen’s identity [59] will be as follows:

(6) $$S{\left(P\right)}_{s,u}^{i_{1},i_{2},\ldots i_{k},\ldots i_{n}}=\sum_{k=0}^{n}S{\left(P\right)}_{s,t}^{i_{1},i_{2},\ldots i_{k}}\;S{\left(P\right)}_{t,u}^{i_{k+1},i_{k+2,}\ldots i_{n}}$$

The length/dimension of path signatures is determined based on the order (*k*) and the degree [41]. Path signature (*S*) of degree 2 was used, which has proven to be useful [60], and looks like this as in Equation (7) for a two-dimensional path, which can be more compact for a log path signature. However, for the two-dimensional path, changing the order to 5 gives a feature vector of 62, and then changing it to 7 gives a feature vector of 254. Therefore, the higher value of k can induce the curse of dimensionality for the traditional ML models.

(7) S={1,S(1),S(2),S(1,1),S(1,2),S(2,1),S(2,2)}

### 2.6. Classification of Fallers vs. Non-Fallers

Six different ML models from various domains such as linear (linear discriminant analysis, logistic regression), nonlinear (SVM, NB, KNN) and tree ensemble (RF) were implemented to evaluate the optimal model to classify fallers from non-fallers. Each model was trained on pre-processed gait characteristics data (standardised, PCA, path signature method) and model performance was compared. Training data for the classifiers were only based on the transformed gait characteristics. To avoid overfitting, we evaluated the models based on 5-fold cross-validation. In every training fold, we utilised 70% of the data, leaving 30% of data in the testing fold (not used in training). This was repeated 5 times with different seed values for randomisation of the data, and results from each fold along with the average are presented. Model performance was evaluated with commonly used evaluation metrics such as the F1 score (harmonic mean of sensitivity and precision), area under the curve (AUC), accuracy, sensitivity and specificity to avoid any misinterpretation of the ML results. For analysis, the SciKit learn python library was used with standard commands under default settings [61].

## 3. Results

### 3.1. Demographics

Table 1 shows participant demographics. In comparison to non-fallers, fallers were older, shorter, weighed less and had lower body mass index (BMI) (all *p* < 0.05).

### 3.2. Differences in Gait Characteristics between Fallers and Non-Fallers

**Figure 2:**
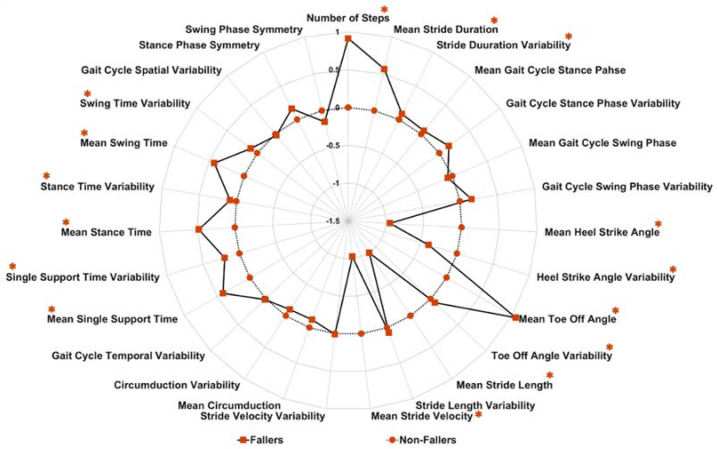
Radar plot indicating the difference between fallers and non-fallers in a range of gait characteristics using z-scores (* indicates significant difference between groups (*p*-value < 0.05)).

**Figure 3:**
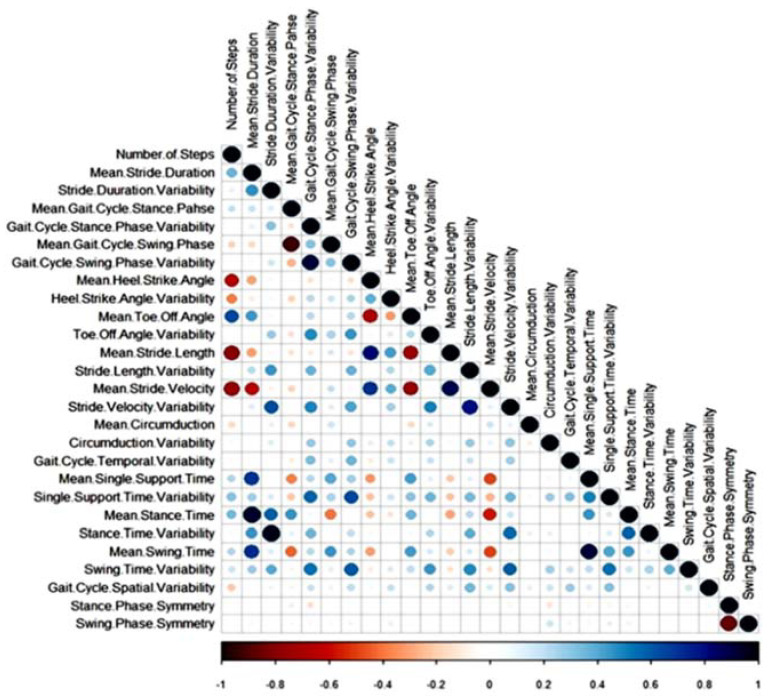
Correlation among the gait characteristics; the bigger the circler, the higher the correlation. Blue means positive correlations and red means negative correlations.

Figure 2 shows the difference in gait characteristics between fallers and non-fallers based on z-scores (difference of CL group mean from PD group mean and divided by the standard deviation of CL group). In comparison to non-fallers, fallers had a slower stride velocity, shorter stride length, reduced heel-strike angle (dorsiflexion), swing phase, swing phase symmetry as well as mean and variability in circumduction values. In contrast, fallers had higher stride length variability, larger mean toe-off angle (plantarflexion), longer stance, swing and single support time variability, longer stance time, a greater number of steps, longer stride duration, increased stride duration variability and stance phase symmetry, compared to non-fallers. The correlation (linear relationship) among gait characteristics was high (Figure 3).

### 3.3. Classification Modelling Results: Combinations of Pre-Processing Techniques and ML Models

**Figure 4:**
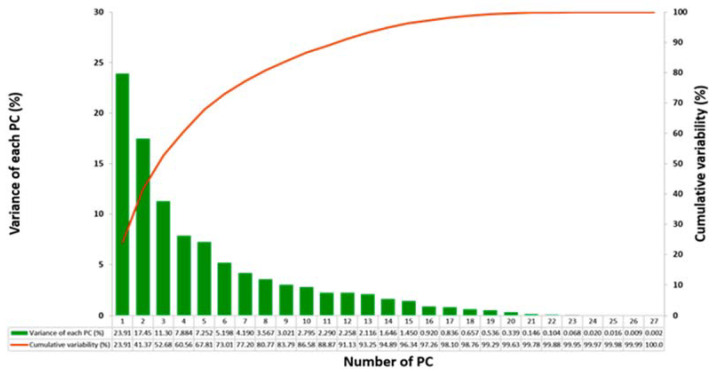
Number of components selected from the principal component analysis (PCA) for training the classifiers.

From PCA (Figure 4), the first five components were selected (eigenvalue > 1), which captured 68% variance of the data. These five components were used for training the ML models. The associated gait characteristics that contributed the most were as follows: swing phase symmetry, stance phase symmetry, mean swing time, mean single support time and mean gait cycle stance phase.

**Figure 5:**
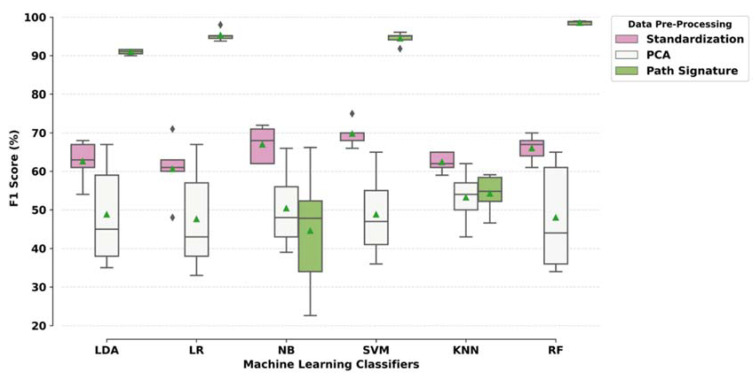
Classification performance of the ML models based on the F1 score. LDA: linear discriminant analysis; LR: logistic regression; NB: Naïve Bayes; SVM: support vector machine; KNN: k-nearest neighbour; RF: random forest.

Classification results from each iteration in the 5-fold cross-validation, where the models were trained on 70% data and tested on the remaining 30%, are given in the Appendix A (Table A1 and Table A2). The average results for these 5 iterations are given in Figure 5 and Table 2. The F1 score was used to select the best performing metric based on the imbalanced dataset. Data pre-processing methods affect classification performance. Overall, the path signature method performed better compared to the other methods. ML models such as RF, SVM, LR and linear discriminant analysis performed better as compared to KNN and NB.

Using standardisation, ML models resulted in a classification accuracy ranging between 61% and 70% with sensitivity of 59–64% and specificity of 62–74%. PCA performed worse than standardisation (z-scores) with classification accuracy of 48–51% with 43–49% sensitivity and 49–56% specificity. The path signature method performed best, boosting the performance of the classifiers with overall accuracy ranging between 63% and 98% with 38–99% sensitivity and 75–98% specificity.

## 4. Discussion

The effect of different data pre-processing methods on the performance of ML models for the optimal classification of neurological patients with and without a history of falls was determined in this study. As hypothesised, data pre-processing methods affected the classification performance of the ML models. The RF model with path signature method could reduce data dimensionality and consider nonlinear interactions among the gait characteristics as well as linear interactions, and gave the most accurate classification of fallers vs. non-fallers. To the best of our knowledge, this is the first study that has examined the application of the path signature as a data pre-processing technique to classify fallers from non-fallers in people with diverse neurological disorders (Parkinson’s disease, stroke, epilepsy, pain syndromes, multiple sclerosis, central nervous system tumour, polyneuropathy, vertigo, dementia and meningitis/encephalitis). The findings from this study suggest that faller classification models trained on gait characteristics pre-processed with the path signature method may be generalised across patient groups with mobility problems.

Six different machine learning models such as LDA, LR, NB, SVM, KNN and RF were selected from linear and nonlinear domains. These models were trained on 27 gait characteristics extracted from a 20 m walk in the neurology ward from patients with a variety of neurological disorders. The accuracy of the classifiers ranged between 48% and 98% with sensitivity of 43–99% and specificity of 48–98%. Overall, RF performed best followed by the SVM (linear), LR, LDA, NB and KNN.

The rationale for selecting different classifiers was to generalise the findings of this study by finding the optimal ML model rather than optimising for a single classifier. Each classifier has its own advantages and disadvantages. Traditional shallow ML models such as SVM do not require a large dataset and can be trained on a reasonably small dataset for reliable estimates of the support vectors that are used for the decision-making function in SVM for classification [62]. However, the interpretability of the SVM model is limited when the number of features are higher (meaning a higher dimensional space) with kernel decision functions instead of linear SVM [63,64]. RF creates multiple decision trees randomly in parallel, by considering the correlation among gait characteristics [65]. RF offers a better interpretability compared to SVM (radial basis function), as it provides information about the most important (i.e., “results and data-driven”) gait characteristics [66]. LDA does not work well when there is an imbalance in classes, and input variables do not follow the multivariate normal distribution [37]. LR works better when independent variables have a good correlation with the dependent or target variables, and is vulnerable to overfitting [67]. NB is based on priors and likelihood, which can be sensitive to skewed data [68]. KNN is a non-parametric algorithm, which is an instance-based learner and influenced by high dimensionality of the data attributes [69]. Apart from traditional ML models, deep learning models require a large amount of training data, and are more suitable for raw sensor data. Therefore, the size of the data and the structured form of dynamic gait outcomes in this study are more appropriate for traditional ML.

There is a lack of research investigating faller classification in patients from a diverse group of neurological conditions [28,70,71]. The classification results from our study were better compared to others. According to Gao et al. [70], model-based and model-free ML techniques were used to classify fallers with Parkinson’s disease using data from different centres. From the Michigan dataset [70], the RF performance ranged between 76% and 77% with sensitivity of 35–44% and specificity of 92–94%. From the Tel-Aviv dataset [70], the RF model performance varied between 69% and 80% with sensitivity of 61–68% and specificity of 79–87%. The maximum accuracy of the RF was 81% with 71% sensitivity and 85% specificity. According to Paul et al. [71], by including fall history, disease severity, leg muscle strength, balance, mobility and freezing of gait, an AUC of 80–83% could be achieved when classifying fallers with a range of neurological disorders.

To improve the classification performance of ML models, data pre-processing was performed in the present study with standardisation, PCA and path signature methods. By standardising the data, model performance varied between 61% and 71% with sensitivity of 59–64% and specificity of 62–74%. With PCA, the model performance ranged between 48% and 51% with sensitivity of 43–49% and specificity of 48–56%. For the path signature method, optimal performance was achieved with 63–98% accuracy, 38–99% sensitivity and 75–98% specificity. Data transformation with pre-processing also helps to reduce multicollinearity among the gait characteristics by extracting new orthogonal features with PCA that are independent of each other and by extracting geometric features with the signature method to reveal the linear and nonlinear interaction in the data. However, the low level of multicollinearity still remains, which is theoretically permissible and may contain useful information for classification. In this study, the model performance was impacted by data transformation, which improved the accuracy of our results.

The main motivation behind standardisation was to rescale the data ensuring a normal distribution to satisfy the assumption of linear classifiers [72]. In addition, standardisation is recommended if the units of features (gait characteristics) are different [50]. Standardisation can help the models (SVM and LR) to update the weights faster and the Euclidean distance measure-based algorithm (KNN) [73]. Tree-based models are unaffected by the different scales of the feature attributes included; however, for important feature selection in RF, it is critical to scale the features [74]. For PCA, it is crucial to standardise the data to capture the variance of the gait characteristics [75]. However, if the covariance structure of the variables are the same, whether standardised or not, they do not affect the PCA [76]. If any variable is scaled with a different covariance, the results of the PCA will be affected [77]. The results from the PCA presented here may be lower than the standardisation method. Since the PCA considered the whole dataset as one class, it is also possible that the number of components used for classification was not appropriate to capture the variance where there was maximum data spread.

The path signature vectors extracted from the rough path characterise the shape of path [41], and have been proven useful in classification tasks where there is a continuous stream of data such as electroencephalography signals [56]. In this study, we considered a stream of 27 gait characteristics to extract signature vectors. The signature vectors provided the exact information required by the ML model to learn the path shape and classify fallers from non-fallers. Interestingly, optimal balance in sensitivity and specificity was achieved with the path signature method, which is critical for the classification of fallers. As a screening tool for fall risk assessment (probability of having future fall; if the probability is greater than 0.50, the patient is a faller and if the probability is less than or equal to 0.5, the patient is a non-faller), high sensitivity with low specificity can result in the identification of more patients who are not at risk of fall who actually have a high risk of falls (false positive). In contrast, with higher specificity and lower sensitivity, the classification tool can result in the identification of more patients who are at high risk of falls who actually are at no risk of a fall (false negative). In a previous study [70], specificity was higher than sensitivity with 71% of sensitivity; there is a 29% chance that PD patients with a high fall risk would be wrongly classified as non-fallers. In the present study, the RF model trained on the signature vectors gave a sensitivity of 99% and specificity of 98%.

### 4.1. Limitations

There are some limitations of the present study. Classifiers were not trained on each subset of neurological disorders such as Parkinson’s disease or dementia, etc. However, models were trained on a group of participants with a variety of neurological disorders to generalise this approach for clinical application. Classifiers were trained on data from fallers identified retrospectively, which acts as the gold standard for labelling the training and testing data for classifiers to predict future falls. Definitions of classifying fallers based on fall history (i.e., a given number of falls within a given time period) vary across the literature [78]. A prior fall is associated with an increased risk of future falls [12,79]; specifically, those who have fallen once are at greatest risk of falling again [46,80,81]. As such, further validation studies are required using prospective data to confirm the findings of the present study. Furthermore, future studies should also investigate and consider the validation issues related to the term “faller” for data labelling. We have demonstrated that it is possible to accurately classify fallers from non-fallers. However, this black box approach does not give clinically meaningful characteristics for proposing appropriate intervention plans. There is a need to identify the underlying and disease-specific impairments, which are linked to increased fall risk. Therefore, in future studies investigating faller classification methods, the underlying fall risk factors should also be included.

### 4.2. Clinical Implications

A fall is a multi-factorial phenomenon and falls often occur while walking [82]. A fall can lead to serious physiological and psychological consequences. Walking impairment assessed with inertial sensors and analysed with ML can help in the proactive fall risk assessment of individuals with neurological disorders. The ML models trained on the gait characteristics of retrospective fallers and non-fallers with neurological disorders gave accurate classification performance. These trained models using retrospective fall data have the potential to assess the future fall risk of patients in a clinical setting based on the extracted gait characteristics from inertial sensors. Already extracted clinically relevant gait characteristics can help to understand the underlying gait impairment for fall risk assessment. This information may support the clinician when proposing tailored intervention plans to prevent future falls. However, the first critical step in this process is to assess fall risk or predict the likelihood of an individual falling with reliable sensitivity and specificity. In the future, clinicians may adopt the approach presented in the present study so that individuals at risk can be identified in advance based on trained models. For any further use of these trained models in clinical practice, it is important that these ML models be further tested on longitudinal datasets to strengthen the proposed feasibility for clinical use and the management of neurological disorders.

## 5. Conclusions

This study explored the effect of three data pre-processing methods on the classification performance of six ML models and proposed a novel application of the path signature method to transform gait data for the optimal classification of fallers with a range of movement disorders. Accurate classification of patients who are at a high risk of falls is possible in the neurological wards to provide appropriate care and intervention plans. Data pre-processing techniques influenced classification accuracy. With the accurate identification of fallers, clinicians can propose tailored interventions for each patient admitted to inpatient clinical settings. We demonstrated that a gait assessment conducted in the clinic using wearable inertial sensors may be analysed with trained ML models using retrospective fall data. Further development of this approach should be applied to identify fall risk prospectively in longitudinal studies and, ultimately, identify fall risk in people who are fall naïve in order to adopt a preventative approach to fall management.

## Figures and Tables

**Table 1:** Demographic characteristics of study participants.

Demographics	Non-Fallers (*n* = 190) Mean ± SD	Fallers (*n* = 159) Mean ± SD	*p*-Value
M/F	115/75	88/71	0.330
Age (year)	61.6 ± 12.2	65.0 ± 12.7	0.009
Height (m)	1.73 ± 0.1	1.70 ± 0.1	0.021
Mass (kg)	81.89 ± 16.35	76.31 ± 14.87	0.002
BMI (kg/m^2^)	27.22 ± 4.76	26.08 ± 4.34	0.027

SD: standard deviation; M: male; F: female; BMI: body mass index. *p*-value < 0.05 considered as statistically significant in independent *t*-test (Age, Height, Mass and BMI) and chi-squared test (M/F).

**Table 2:** Accuracy, sensitivity and specificity of ML models.

ML Models	Data Pre-Processing Methods Accuracy (Sensitivity, Specificity)%		
	Standardisation	PCA	Path Signature
Linear Discriminant Analysis (LDA)	63.10(62, 62)	48.95(45, 52)	91.81(91, 92)
Logistic Regression (LR)	60.75(59, 62)	47.81(44, 51)	95.80(97, 95)
Naïve Bayes (NB)	67.81(64, 68)	51.24(51, 48)	63.05(38, 82)
Support Vector Machine (SVM-linear)	70.48(64, 74)	50.67(43, 56)	95.05(95, 95)
K-Nearest Neighbour (KNN)	62.28(61, 63)	50.28(49, 52)	63.04(49, 75)
Random Forest (RF)	66.28(63, 69)	48.19(49, 49)	98.67(99, 98)

## Appendix A

**Table A1:** ML model results for accuracy (sensitivity, specificity).

**Results Based on Standardised Spatial-Temporal Features** **Accuracy (Sen, Spec)**						
**ML Models**	**Split 1**	**Split 2**	**Split 3**	**Split 4**	**Split 5**	**Average**
LDA	68.57(0.79, 0.56)	61.9(0.49, 0.73)	63.81(0.51, 0.76)	53.33(0.57, 0.51)	67.9(0.76, 0.55)	63.10(0.62, 0.62)
LR	71.43(0.79, 0.63)	63.8(0.49, 0.77)	61.9(0.51, 0.72)	47.62(0.54, 0.42)	59(0.62, 0.55)	60.75(0.59, 0.62)
NB	62.86(0.76, 0.49)	71.43(0.55,0.86)	62.86(0.47, 0.78)	72.38(0.57, 0.85)	69.52(0.86, 0.43)	67.81(0.64, 0.68)
SVM	70.48(0.87, 0.53)	68.57(0.53,0.82)	67.62(0.47, 0.87)	70.48(0.52, 0.85)	75.24(0.83, 0.63)	70.48(0.64, 0.74)
KNN	64.76(0.72,0.57)	60.95(0.59, 0.63)	61.9(0.55, 0.69)	64.76(0.54, 0.73)	59.05(0.63, 0.53)	62.28(0.61, 0.63)
RF	63.8(0.72,0.55)	70.48(0.61, 0.79)	61.9(0.51, 0.72)	67.62(0.59, 0.75)	67.62(0.71, 0.63)	66.284(0.63, 0.69)
**Results based on PCA** **Accuracy (Sen, Spec)**						
**ML Models**	**Split 1**	**Split 2**	**Split 3**	**Split 4**	**Split 5**	**Average**
LDA	60(0.76, 0.43)	67.62(0.51, 0.82)	38.1(0.31,0.44)	44.76(0.35, 0.53)	34.29(0.32, 0.38)	48.95(0.45, 0.52)
LR	58.1(0.7, 0.45)	67.62(0.51, 0.82)	38.1(0.33, 0.43)	42.86(0.39, 0.46)	32.38(0.29, 0.38)	47.81(0.44, 0.51)
NB	58.1(0.78, 0.37)	66.67(0.49, 0.82)	39.05(0.31, 0.46)	42.86(0.33, 0.51)	49.52(0.65, 0.25)	51.24(0.51, 0.48)
SVM	58.1(0.85, 0.29)	67.62(0.41, 0.91)	42.86(0.26, 0.59)	49.52(0.24, 0.7)	35.24(0.39, 0.3)	50.67(0.43, 0.56)
KNN	55.24(0.69, 0.41)	61.9(0.57, 0.66)	45.7(0.24, 0.67)	39.05(0.44, 0.36)	49.52(0.51, 0.48)	50.28(0.49, 0.52)
RF	60.95(0.69, 0.53)	64.76(0.59, 0.7)	36.19(0.41, 0.32)	43.81(0.52, 0.37)	35.23(0.25, 0.53)	48.19(0.49, 0.49)
**Results based on Path Signature Method** **Accuracy (Sen, Spec)**						
**ML Models**	**Split 1**	**Split 2**	**Split 3**	**Split 4**	**Split 5**	**Average**
LDA	91.43(0.961,0.87)	91.43(0.94, 0.89)	90.47(0.88, 0.93)	92.38(0.83, 1)	93.33(0.95,0.923)	91.81(0.91, 0.92)
LR	95.24(0.98, 0.93)	98.095(1,0.96)	95.2(0.96, 0.94)	95.24(0.94, 0.97)	95.24(0.95,0.953)	95.80(0.97, 0.95)
NB	54.28(0.137,0.93)	66.67(0.33, 0.96)	57.14(0.86, 0.29)	70.47(0.37, 0.97)	66.67(0.23, 0.94)	63.05(0.38, 0.82)
SVM	95.24(0.96, 0.94)	96.19(1, 0.93)	94.28(0.94, 0.94)	93.33(0.85, 1)	96.19(1, 0.94)	95.05(0.95, 0.95)
KNN	64.76(0.51, 0.78)	65.71(0.53, 0.77)	58.09(0.47, 0.69)	63.810(0.50,0.75)	62.857(0.43,0.75)	63.04(0.49, 0.75)
RF	99.05 (1,0.98)	98.09 (1,0.96)	98.09 (0.98, 0.98)	99.05(0.98, 1)	99.05 (1, 0.98)	98.67(0.99, 0.98)

**Table A2:** ML model results for F1 Score and AUC.

**Results Based on Standardised Spatial-Temporal Features** **F1 Score (AUC)**						
**ML Models**	**Split 1**	**Split 2**	**Split 3**	**Split 4**	**Split 5**	**Average**
LDA	0.68(0.71)	0.61(0.7)	0.63(0.63)	0.54(0.54)	0.67(0.67)	0.63(0.65)
LR	0.71(0.7)	0.63(0.69)	0.61(0.63)	0.48(0.49)	0.6(0.63)	0.61(0.63)
NB	0.62(0.66)	0.71(0.76)	0.62(0.65)	0.72(0.74)	0.68(0.67)	0.67(0.7)
SVM	0.7(0.7)	0.68(0.68)	0.66(0.67)	0.7(0.69)	0.75(0.73)	0.7(0.7)
KNN	0.65(0.67)	0.61(0.59)	0.62(0.65)	0.65(0.64)	0.59(0.62)	0.62(0.63)
RF	0.64(0.7)	0.7(0.74)	0.61(0.64)	0.67(0.72)	0.68(0.76)	0.66(0.71)
**Results based on PCA** **F1 Score (AUC)**						
**ML Models**	**Split 1**	**Split 2**	**Split 3**	**Split 4**	**Split 5**	**Average**
LDA	0.59(0.62)	0.67(0.73)	0.38(0.34)	0.45(0.41)	0.35(0.31)	0.49(0.48)
LR	0.57(0.62)	0.67(0.73)	0.38(0.34)	0.43(0.41)	0.33(0.30)	0.48(0.48)
NB	0.56(0.60)	0.66(0.71)	0.39(0.35)	0.43(0.37)	0.48(0.39)	0.50(0.48)
SVM	0.55(0.57)	0.65(0.66)	0.41(0.42)	0.47(0.47)	0.36(0.34)	0.49(0.49)
KNN	0.54(0.57)	0.62(0.65)	0.43(0.47)	0.57(0.56)	0.50(0.50)	0.53(0.55)
RF	0.61(0.65)	0.65(0.67)	0.36(0.35)	0.44(0.42)	0.34(0.35)	0.48(0.49)
**Results based on Path Signature Method** **F1 Score (AUC)**						
**ML Models**	**Split 1**	**Split 2**	**Split 3**	**Split 4**	**Split 5**	**Average**
LDA	0.916(0.916)	0.911(0.916)	0.900(0.904)	0.905(0.913)	0.916(0.937)	0.909(0.917)
LR	0.952(0.953)	0.980(0.982)	0.951(0.953)	0.945(0.950)	0.938(0.952)	0.953(0.958)
NB	0.226(0.532)	0.478(0.645)	0.662(0.580)	0.523(0.668)	0.340(0.582)	0.445(0.601)
SVM	0.951(0.953)	0.961(0.964)	0.941(0.943)	0.918(0.924)	0.952(0.969)	0.945(0.951)
KNN	0.584(0.644)	0.591(0.649)	0.522(0.578)	0.548(0.623)	0.466(0.589)	0.542(0.616)
RF	0.990(0.991)	0.980(0.982)	0.980(0.981)	0.989(0.989)	0.988(0.992)	0.985(0.987)

## Appendix B

In the current study, the gait parameters were extracted based on the proprietary algorithms from Hasomed (RehaGait, Magdeburg, Germany). Other published studies using RehaGait system can be seen here: https://hasomed.de/en/company/references/#rehagait. The description of parameters is as follows:

- Stride duration: duration of a stride (in seconds)
- Stride length: length of a stride (in meters)
- Stride velocity: velocity of a stride (in meters per second)
- Number of steps: number of steps during the test
- Stance and swing phase duration (as percent of gait cycle)
- Stance and swing time (in seconds)
- Symmetry of stance and swing phases: symmetry calculated based on time differences between the respective left and right phases for a given outcome
- Single support time: time at which only one foot is on the floor (in seconds)
- Heel-strike angle and toe-off angle: angle of the forefoot during the specific time points (in °)
- Foot circumduction: foot circumduction describes the swing width (distance between an imaginary straight walking line of foot and the point of maximum actual deflection of the respective foot during the swing phase) of the respective leg (in meters)

## Appendix C

The path for signature extraction data for one faller and one non-faller participant are included below (see Figure A1), which are based on the extracted gait characteristics that are arranged to make a piecewise linear path; the axes of the figure are unitless and dimensionless.
